# Health and Hell

**Published:** 1889-07

**Authors:** S. H. Preston


### HEALTH AND HELL.

BY S. H. PRESTON.

No. 3.

Two miles out from Geneva was a grand elevation of ground called Champel. All around, as far as the eye could see, was spread to view the charming and contrasted scenery of Switzerland—verdant valleys and vine-clad hills, the flashing waters of the Rhone and winding Arve, the evergreen, pine-crowned peaks of the Jura, and the glistening glaciers of Savoy.

Here on the morning of October 27, 1553, had collected a great crowd of people. A stake is firmly fixed in the earth, to which, upon a block beside it, is chained a man of lofty and intellectual mien. Fagots are piled about his feet, upon which are heaped green oak logs with leaves still hanging to them.

The torch is touched. The flames flash fiercely up at first, but the green oak smothers the fire. The executioners cruelly stir the coals and cast on more wood. This simmers and crackles while the sufferer slowly roasts to death. At last, after long and terrible torment, the welcome flames leap up and wrap his writhing form and white, heroic face, and erelong all that is left of him is a charred and smoking carcass.

Thus was the idea of divine retribution exemplified on earth, thus was a heretic hurried to hell in the name of John Calvin's God. This God burns the souls of men in eternal fire for their erronious beliefs. We cannot serve him better than by abolishing those beliefs ; and the best way to do that is to burn the bodies of those that propagate them. Nothing more delights the nostrils of this God than the smell of a burning heretic. And it is really a kindness to the heretic to burn him, because it cuts him off from further adding to his own damnation. It also may prevent many precious souls being led astray by him, their damnation serving to swell his own. Even prolonging his agony by burning him slowly as possible is for his benefit, as it affords him further time for abjura ion and repentance. Of course it seems cruel, as does the severing of a limb by a surgeon to save life, but the pious purpose is to prevent punishment in everlasting fire. So that there is really an excellent economy of suffering in the long run.

This heretic consumed in the green oak fire at Champel was a scientific Spaniard named Michael Servetus. His crime was believing in one God instead of three, and being too great a man for his time. From Aragon he had gone to Toulouse, where he adopted the profession of an advocate. Thence he went up to Paris, the great capital that has both coddled and killed so many of the children of genius. There he studied medicine and obtained the diploma of a doctor. Another thing he did, and note it well. *He published an elaborate treatise on the circulation of the blood.* The fact that this learned heretic wrote a work on the circulation of the blood long before Harvey lived should never be forgotten. Both the printed and manuscript copy of this remarkable book, together with his religious writings, were bound about his neck and burned with him at the stake.

A man with his progressed opinions could not long live in peace in Paris. So he fled to Lyons, from there to Vienne on Dauphine, where he was arrested, and thence escaped to the Protestant city of Geneva. Human bloodhounds were everywhere upon his track because he believed in one God instead of three. Now, at Geneva was another fugitive reformer and heretic, a surly man with a sour stomach and a stone in his bladder, who had been banished from the city and recalled. This was John Calvin, a strenuous stickler for three Gods instead of one.

Ingersoll likens the escape of Servetus to the city of Calvin to the flight of a dove from hawks right into the nest of a vulture. Had the great agnostic been as well informed respecting Calvin as of the "Mistakes of Moses," he would not himself have made the monstrous mistake of charging him with Servetus' death, and of venting his virulent volubility upon his voiceless dust for such an assumed atrocity. It avails little to refute this stereotyped and cruel charge; for fast as each fledgeling infidel discards the Presbyterian old-day doctrines he shouts in gloative glee, "But Calvin burnt Servetus." The absurdity of this ancient accusation will be apparent from the following facts.

Geneva was an independent Republican city of 20,000 population. Its municipal government, established long before Calvin came to it, consisted of a Council of Two Hundred, which alone could enact and annul laws. A lesser Council of Sixty was principally for diplomatic purposes. There were four Syndics, or civil magistrates, with their Council of Sixteen. This body had power of life and death. Twice a year there were sessions of the General Assembly. Nothing was brought before this body which had not been acted upon by the Two Hundred, and nothing by them that had not passed through the Council of Sixty, and nothing by them that had not been approved by the Council of Sixteen.

The Consistory was simply a spiritual body composed of elders and one member of the civil court. It possessed no temporal power whatever. The elders were elected by the great council and the citizens. The church was completely under the control of the city government, its preachers being paid by the city instead of the people, and a civil judge presiding in its synods. So that the clergy had not the slightest show of civil authority. The church was entirely under lay government. Let this be borne in mind when we are told of the terrible theocracy established by Calvin.

Calvin sought safety in Geneva from his religious foes at the age of twenty-eight. Through the influence of his friend, Farel, he was appointed a teacher of theology, and erelong elected preacher by the magistrates and people. But the citizens soon became displeased with his rigid ways and stern reproofs. He did endeavor to dictate to them regarding what he deemed their demoralizing amusement, and the result was that those liberty loving Genevans, who still defied their ducal sovereign of Savoy in defence of their personal privileges, ill-disposed to brook the interference of a fugitive Frenchman, banished both Calvin and Farel. After an absence of three years his offence was condoned by his congregation, who called him back and gave him a cloth cloak, as he was not comfortably clad.

And Ingersoll would have us believe that this religious refugee sneaked in among those valiant Swiss that had battled for liberty since the time of Tell, who overturned the Austrian tyranny upon the fierce-fought field of Morgarten, whose army of 1,600 men chose to be cut to pieces on the field of St. Jacques sooner than surrender to 20,000 French soldiers, and whose infantry was still the terror of Europe—that this proscribed preacher robbed that patriotic people of their rights, that this banished man usurped the government of Geneva, banished its chief citizens, beheaded their poet, John Gruet, burnt Servetus, and outdid Nero there for a generation. The Presbyterian creed may be a bad concern, but in our deprecation of it, there is no need to falsify the facts of history concerning Calvin, who, as we have shown, did not originate it.

The extent of Calvin's terrible tyranny may be inferred from the fact that his yearly salary was $50, beside house rent and a small allowance of corn and wine. This scarcely sufficed for the necessaries of life, and he went days without eating. After the death of his wife, who for nine years proved a real helpmate to him, he found that he could not afford to marry again. He abode in a poor tenement, and his clothes were always coarse and cheap. His total property at his death was less than $200. If he was such a despotic monster as Ingersoll alleges, he surely was not a mercenary one.

Now as to the burning of Servetus. His crime was heresy, in that age regarded as much more heinous than murder as murder is than misdemeanor to-day. The murderer only killed the body, but the heretic assassinated the soul. Calvin was merely a teacher of theology at Geneva, and the clergy had no hand in the administration of civil affairs. What Ingersoll miscalls Calvin's Consistory possessed no more power for punishment than a Presbyterian Conference. In that century, the crime of Servetus was one against the state, and he was condemned by the judiciary of Geneva and put to death by the secular authorities. This would have happened had Calvin not been in the city. It is a question whether Calvin could have saved Servetus, and another whether he would have saved him if he could. The worst that can be charged against Calvin is that he acquiesced in the sentence. But for that he can be no more justly blamed than Sir Matthew Hale for sentencing witches or our Colonial ancestors for hanging them.

And Servetus could have saved himself. He had only to say, "I believe in the Son of the Eternal God," instead of speaking of Christ as the "Eternal Son of God." Only the difference between the Trinitarian and Unitarian arrangement of an adjective. And for aught that appears to the contrary, Servetus would have burnt Calvin had he been offered the opportunity. A person who will persistently provoke persecution for his religious opinions will persecute others for opposite opinions. A man who will endure death for his doctrine will endeavor to enforce that doctrine unto death. This fact has blurred with blood the history of Christianity.

While Ingersoll is so eloquently clamoring for justice to Thomas Paine let him accord the same to John Calvin.

That this installment is not quite pertinent to the title of the series, is due to our great desire to clear the mud of malice from the memory of the most maligned man that ever lived.

504 GRAND STREET, NEW YORK, May 14, 1889.

*To the Editor of* HALLS JOURNAL OF HEALTH :

*Dear Sir.*—I enclose a paper entitled "Health without Medicine" which I hope you may like. It has at least one merit, that of being a literal statement of facts.

Yours truly,

THEODORE H. MEAD.

